# Effects of a 6-week integrated dementia prevention intervention in community-dwelling older adults

**DOI:** 10.1093/geroni/igab046.3063

**Published:** 2021-12-17

**Authors:** Sunghee Tak, Ji-yeon Kim, Hana Ko, SuJung Jung, Jaegyeong Lee

**Affiliations:** 1 Seoul National University, Seoul, Seoul-t'ukpyolsi, Republic of Korea; 2 Eulji University, Uijeongbu, Seoul-t'ukpyolsi, Republic of Korea; 3 Gacheon University, Incheon, Seoul-t'ukpyolsi, Republic of Korea; 4 Semyung University, Jecheon, Seoul-t'ukpyolsi, Republic of Korea

## Abstract

This study examined the week of a 6-session integrated dementia p program on dementia knowledge, attitude toward dementia, fear of dementia, and dementia prevention behaviors among community dwelling elders. Using a nonequivalent control group, pre-posttest design, study participants were recruited from a senior center in Seoul, Korea. A total of 40 participants completed the study while half of them were in the experimental group and another half were in the control group. They completed survey questionnaires before and after the program. The findings showed that the program was effective only to decrease the fear of dementia of the older adults. Knowledge, attitude toward dementia, and healthy behaviors may be difficult to change in a short period of time. However, the integrated dementia prevention program may be effective to decrease negative emotions, particularly, fear toward dementia among older adults. The fear of dementia needs to be further assessed individually in order to identify particular causes and triggers and provide tailored interventions.

